# Correlation Between Skin and Affected Organs in 52 Sclerodermic Patients Followed in a Diseases Management Team: Development of a Risk Prediction Model of Organ-Specific Complications

**DOI:** 10.3389/fimmu.2021.588753

**Published:** 2021-06-02

**Authors:** Emanuele Cozzani, Andrea Muracchioli, Giuseppe Murdaca, Mirko Beccalli, Simone Caprioli, Patrizia Zentilin, Pietro Ameri, Marco Grosso, Rodolfo Russo, Luca Carmisciano, Aurora Parodi

**Affiliations:** ^1^ Dermatologic Unit, University of Genoa, DiSSal, Ospedale-Policlinico San Martino, IRCCS, Genova, Italy; ^2^ Departments of Internal Medicine, Scleroderma Unit, Clinical Immunology Unit, University of Genova, Ospedale-Policlinico San Martino, IRCCS, Genova, Italy; ^3^ Department of Healt Sciences DiSSal, University of Genova, Ospedale-Policlinico San Martino, IRCCS, Genova, Italy; ^4^ Division of Gastroenterology, Department of Internal Medicine, University of Genova, Ospedale-Policlinico San Martino, IRCCS, Genova, Italy; ^5^ Cardiovascular Disease Unit, IRCCS, Ospedale-Policlinico San Martino; Department of Internal Medicine, University of Genova, Genova, Italy; ^6^ Interventional Pneumology Unit, Ospedale-Policlinico San Martino, IRCCS, Genova, Italy; ^7^ Nephrology, Dialysis, and Transplantation, Ospedale-Policlinico San Martino, IRCCS, Genova, Italy

**Keywords:** systemic sclerosis, scleroderma, mRSS (Rodnan Score), predictive model, organ complications

## Abstract

**Objective:**

To identify the existence of a correlation among the various organs affected, focusing primarily on immuno-dermatological aspects, and to create a risk prediction model of organ-specific complications.

**Material and Methods:**

Fifty-two patients with stable scleroderma, followed between 2015 and 2019, were investigated through an extensive multidisciplinary evaluation in the last year.

**Results:**

Patients with lung involvement presented a worse degree of skin fibrosis than patients without it (p <0.001). No relationship was observed for the heart, kidney, and esophagus. Patients with pulmonary involvement had a lower pressure of the low esophagus sphincter and a higher Warrick score than patients without it (p <0.05). Age was significantly higher in patients with kidney involvement. Diffuse scleroderma patients had a worse pulmonary impairment than limited scleroderma patients (p <0.05). The manometric “sclerodermic” pattern was observed to be the most frequent (55.6%, p <0.05) in dcSSc patients while the sclerodermic and normal pattern were equally represented (41.2 and 32.4% respectively, p <0.05) in lcSSc patients. When compared to the negative serological groups, anti-Scl-70 positive patients presented a worse lung involvement while anti-centromere patients presented a better lung outcome (p <0.05). PM-Scl 100/75 positive patients presented mostly a pulmonary fibrotic pattern (p <0.05) and, also, heart complications were more likely associated with anti PM-Scl 100/75 positivity (p <0.05). The risk prediction model for organ-specific complications had an accuracy of 84.4% (95%CI 78, 89) in complication-site prediction, AUC of 0.871, 86% of sensitivity, and 83% of specificity, Cohen’s Kappa (k) of 0.68.

**Conclusions:**

Out of all the organs studied, the skin is the one that correlates with the lung. Patients with a diffuse form of disease presented more frequently the anti Scl-70 antibody and had a worse lung and esophageal involvement (scleroderma pattern) than the negative group. Conversely, patients with limited disease presented all positive for the anti-centromere antibody with a better lung involvement than the negative group, without any difference among the esophageal manometric pattern. Anti PM-Scl 100/75 antibody patients were associated with pulmonary fibrosis and presented cardiac involvement. The model created has demonstrated excellent values of sensitivity, specificity, and accuracy, but further studies are needed for validation.

## Introduction

Systemic sclerosis (SSc) is an immune-mediated disease that affects, in various degrees of severity, the skin and organs such as the heart, kidney, lung, and esophagus. Over the years, the clinical manifestations, the severity with which the disease affects individual organs, and the course of complications have been studied and described in detail. Correlations between different organs have been reported through the most varied diagnostic methods, demonstrating, in several cases, the existence of progressive interconnected organ damage. Background of previous research: the association among the severity of different organs in scleroderma patients have been described in the literature: most of these concerns the relationship between two organs, especially the esophagus and lung. Moreover, the correlation between skin thickness score and quantitative measurements of each affected organ has also been described. This is the first study that directly proposes a correlation between the severity of skin involvement and quantitative measures of organ involvement. Our model is focused, using immediate parameters to obtain, the probability that a particular organ is affected at the time of the initial assessment. In this way, an individual risk rank of organ damage is profiled (for example lung > esophagus > kidney > heart) thus directing the patient to the reference specialist.

The aim of this study was (1) to identify possible cross-relationships between the various affected organs, and (2) develop a risk prediction model for organ-specific complications, based on clinical and dermato-immunological data, to identify prematurely those patients who may develop organ damage.

## Material and Methods

Fifty-two patients with scleroderma followed in our Scleroderma Unit by Cardiologists, Dermatologists, Gastroenterologist, Immunologists, Pulmonologist, Nephrologist, between 2015 and 2019 were evaluated in the last year. The patients recruited had stable disease (at least 4 years from diagnosis) and underwent an extensive medical assessment, with standardized reporting of history, physical evaluation, and laboratory investigations. Thirty-five out of 52 patients were in therapy. Twenty-two were in monotherapy (eight patients with ACE-inhibitors, seven with mycophenolate mofetil, four with methotrexate and three with bosentan), and 13 in polytherapy (four patients with mycophenolate mofetil and bosentan, three patients with mycophenolate mofetil and ACE-inhibitors, two patients with mycophenolate mofetil, bosentan and ACE-inhibitors, two patients with methotrexate and bosentan, one patient with methotrexate and ACE-inhibitors, one patient with ACE-inhibitors and bosentan). Furthermore, all patients received therapy with prostanoids.

Demographic information regarding age and sex was collected. Disease duration was defined as the interval between the first manifestation of SSc (whether Raynaud’s phenomenon or other presentations) and the baseline study visit. Infusion delay was considered as the years from diagnosis to the start of prostaglandin infusions. All patients fulfilled the diagnostic criteria of the 2013 American College of Rheumatology/European League Against Rheumatism. Patients were divided into two groups according to the extent of skin involvement: limited (lcSSc) and diffuse scleroderma (dcSSc). In addition, the concomitant presence of overlapping diseases, including SLE, SS, rheumatoid arthritis, PM/DM, and/or mixed connective tissue disease was investigated and excluded. All subjects provided a written informed consent to participate in the data collection protocol. For each patient, the following investigations were performed:

### Dermatologic Evaluation

The severity of skin involvement was assessed by using the modified Rodnan skin score (mRSS), a semi-quantitative test that assesses the plicability and thickening of the scleroderma skin. The “Representative area technique” was used for the evaluation of 17 body areas (hand, forearm, arm, foot, leg, thigh, abdomen, chest, and face) assessing the skin thickening with scores ranging from 0 (no involvement) to 3 (severe thickening) (total score range 0–51) (1).

Limited cutaneous SSc (lcSSc) was defined as a skin involvement distal to the elbows and knees, with or without facial involvement, while diffuse cutaneous SSc (dcSSc) was defined as a skin involvement proximal to the elbows and knees, with or without truncal involvement (2). Those with a clinical diagnosis of SSc but no skin involvement (i.e. systemic sclerosis sine scleroderma) were excluded.

### Serology

Using a standardized operating protocol, serum for autoantibody analyses was collected. Anti-nuclear antibodies (ANA) positivity was detected by the indirect immunofluorescence test (IIF) performed on the HEp2 cell line (EUROIMMUN Lübeck, Germany) for dilutions above 1:80 (data not shown). Antibodies against extractable nuclear antigens (ENA) such as anti-Scl70, anti-RNP, anti-SSA/Ro, and specific scleroderma-associated antibodies such as anti-centromere, Anti-RNA polymerase III, Anti PM-Scl 100/75 were detected by ELISA enzyme immunoassay (Orgentec Mainz, Germany).

### Gastroenterological Evaluation

Esophageal impairment has been evaluated by high-resolution esophageal manometry (HRM) according to Savarino et al. (3, 4). A solid-state manometry 32 solid-state sensors spaced at 1-cm intervals (O.D. 4.2 mm) was used (Sandhill Scientific Instruments Inc., Highlands Ranch, CO, USA). Studies were done in the supine position after at least a 6-h fast (4). Motor alterations of the esophagus body (BODY) were evaluated through various manometric parameters and the distal waves amplitude (normal range: 30–180 mmHg) is the main considered. Furthermore, we assessed the pressure at the lower esophageal sphincter (LES), dividing patients into two categories: physiological LES group (10–45 mmHg) and hypotonic LES group (<10 mmHg). The esophagus impairment was considered positive in the following cases: a) when LES was hypotonic (<10 mmHg); b) when BODY presented an ineffective peristalsis (<30 mmHg); c) when BODY presented an ineffective peristalsis (<30 mmHg) and a normotonic LES (10–45 mmHg), d) when LES was hypotonic (<10 mmHg) and BODY presented an ineffective peristalsis (<30 mmHg)

### Pulmonary Evaluation

Functional lung involvement was assessed through the execution of Spirometry and alveolus capillary carbon monoxide diffusion (DLCO), using a large flowmeter (SensorMedics-Viasys, CareFusion; Höchberg, Germany). Single breath, alveolus capillary carbon monoxide diffusion (DLCO) values were measured and adapted to blood concentrations of hemoglobin and carbon monoxide (MasterScreen PFT System, Jaeger-Viasys, CareFusion) according to ATS/ERS guidelines (5). A DLCO cut-off <75% was chosen to distinguish patients with and without functional lung involvement. The forced expiratory volume in one second (FEV1), total lung capacity (TLC) and forced vital capacity (FVC) were also recorded and were considered abnormal if ≤80% of predicted values (6, 7). Possible anatomic involvement of the lung was assessed by performing a high-resolution CT (HRCT). Spirometry data were then compared with the Warrick’s score, a semi-quantitative score obtained from pulmonary CT scans, performed by the same radiologist. Elementary lesions considered in the score and rated from 1 to 5 according to severity were documented (8). The scores for lesion severity and extent were combined, to provide a total HRCT score that ranges between 0 and 30. A cut-off score of 7 was required to consider HRCT abnormalities in SSc as predictive of pulmonary involvement (HRCT score ≥7: positive; HRCT score 7 <: negative) (8).

### Cardiological Assessment

Trans-thoracic echocardiography was performed focusing on the right heart (9). Right ventricular (RV) end-diastolic basal diameter, tricuspid annular plane systolic excursion (TAPSE), Tissue Doppler S’ peak velocity, and fractional area change, and tricuspid regurgitation (TR) was assessed in the apical four-chamber view. RV systolic pressure was calculated by TR peak velocity using the simplified Bernoulli equation. The TAPSE/PASP ratio, an index proposed as RV function evaluation, was calculated and a cut-off ≥0.64mm/mmHg was used to identify patients with or without initial heart involvement (10).

### Kidney Evaluation

The Doppler-derived renal resistive index (R.R.I) was used to detect changes in intrarenal perfusion after echography excluded renal artery stenosis. A cut-off value of ≥0.70 mm/mmHg (as in, the highest between the two kidneys values) has been used to identify patients with possible perfusion impairment due to aging or secondary to renal vascular disease (11). In addition, it was reported how many patients were in therapy with ACE-inhibitors in the period studied.

### Statistical Analysis

Mean, Median, Standard deviation (SD), and Inter Quartile Range (IQR) were used to summarize continuous variables; counts, and percentages to summarize categorical ones. T-test and Mann–Whitney test were used to detect differences in continuous variables between two groups, and the chi-squared test was used for categorical variables Multinomial logistic regression was used to estimate the risk to develop organ-specific complications. Cohen’s Kappa (k) was used to evaluate model performance, quantifying the association between the predictions and the observations, overall and within complication sites. For each organ, a 2 × 2 contingency table has been created to define the number of true positives (TP), false positives (FP), true negatives (TN), and false negatives (FN) predicted by the model. The results were also reported through ROC (Receiver operating characteristic) curves overall and for each organ. For each ROC, the area under the curve (AUC) has been calculated.

No train-validation model development was used because of the small sample size and the explorative nature of this study. P-values below 0.05 were considered significant. R software version 3.6.0 was used for all statistical analyses.

### Predictive Model

Multinomial logistic regression was used to estimate the risk to develop organ-specific complications for each investigated complication (esophagus, heart, lung, and kidney). The multinomial logistic regression is a classification method that generalizes the binary logistic regression to multiclass problems (with more than two possible mutually exclusive discrete outcomes); it is used to predict the probabilities of the different possible outcomes.

We used the complication occurrence as a dependent variable (presence or absence of each complication) and the generic (age, disease duration, infusion delay) and immuno-dermatological (antibodies positivity, Rodnan score) characteristics, routinary available at the dermatologic visit, as independent variables (predictors).

Multinomial logistic regression allowed to simultaneously estimate the probability to occur of each outcome class (presence of esophagus, heart, lung, and kidney complication) based on the set of predictors, in a similar manner of the binomial logistic regression allowing to estimate the probability to occur of each one complication at a time.

The variable selection was performed manually based on clinical criteria: usual availability at the dermatologic visit. Thus, all the clinical information, specific to detect each organ-specific complication (such as Warrick’s score, TAPSE/PASP ratio, possible kidney perfusion impairment, manometric parameters of the esophagus) usually not available at the dermatologic visit, were not included in the predictive model.

The purpose of modelling was to demonstrate the feasibility of estimating the organ-specific complications more likely to occur with a specific set of patients ‘characteristics, using the output probability of having each outcome class (probability of esophagus, heart, lung, and kidney complication) estimated by the model. This is the first step to develop future tools for the tailored complication predictions for every single individual.

To simplify, we assumed that the most frequently observed complication was also the most likely to happen at any moment within the available follow-up (no time-dependent analysis), and we fitted the model using only the patients with all the predictors available (complete-case analysis).

The model was validated internally, and we reported the metrics of the goodness of fit, where for each patient we first estimated the most likely to occur complication and then compared these predictions with the observed complications (Cohen’s Kappa, FN, FP, TN, TP, ROC and AUC).

To describe and report the statistical modelling, we followed the transparent reporting of a multivariable prediction model for individual prognosis or diagnosis (TRIPOD) (12).

Together with the code used for this study, the applied methodology is described and documented in a step-by-step procedure, available as open-source in a public repository [https://github.com/pqstri/SSc/blob/main/ssc.R], to extend the reproducibility and the generalizability of the methodology and to allow replication in other studies.

## Results

**Figure 1:**
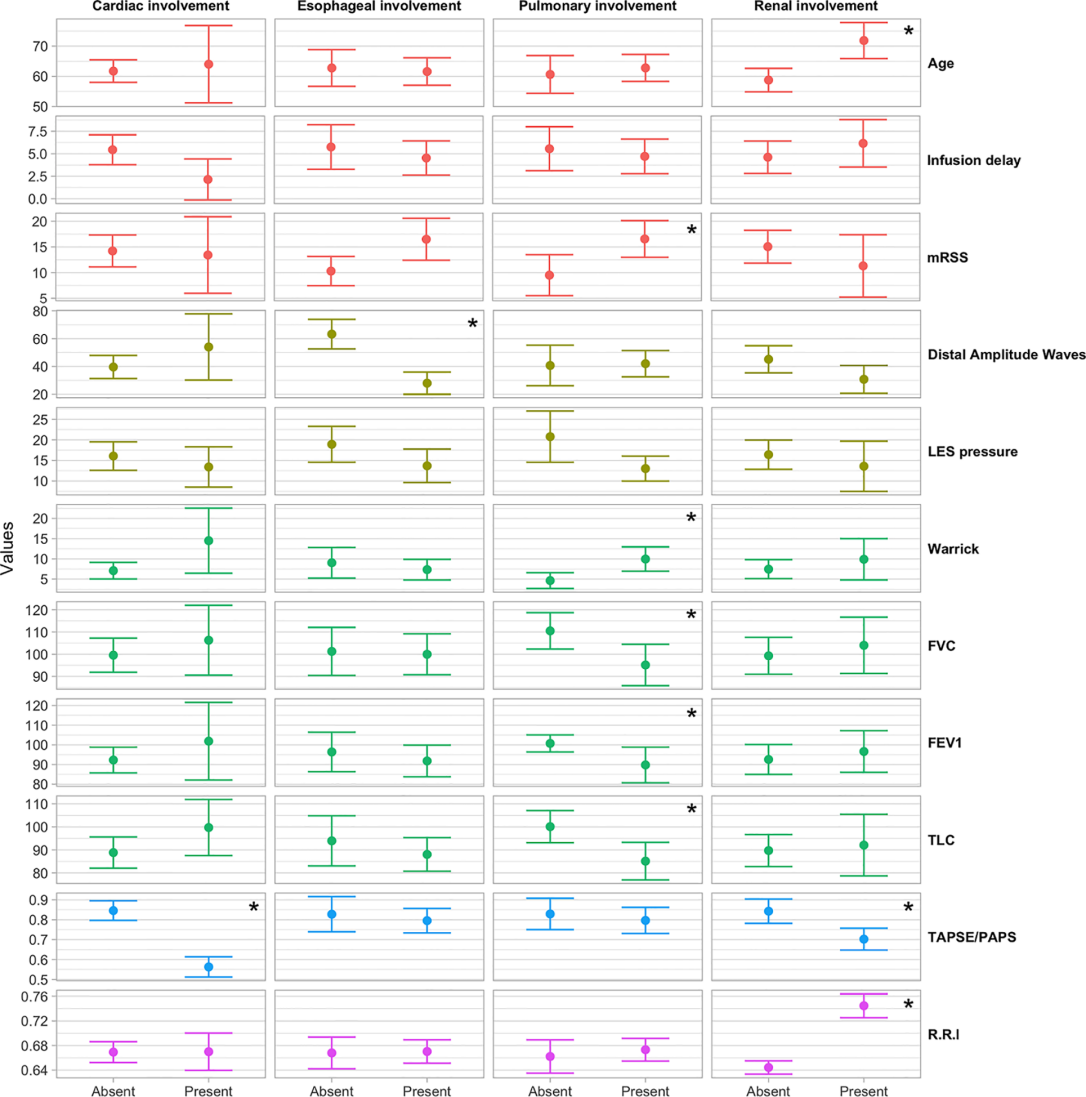
Box plot of the distribution of instrumental parameters by the occurrence of heart, esophagus, kidney and pulmonary involvement. *p < 0.05.

**Figure 2:**
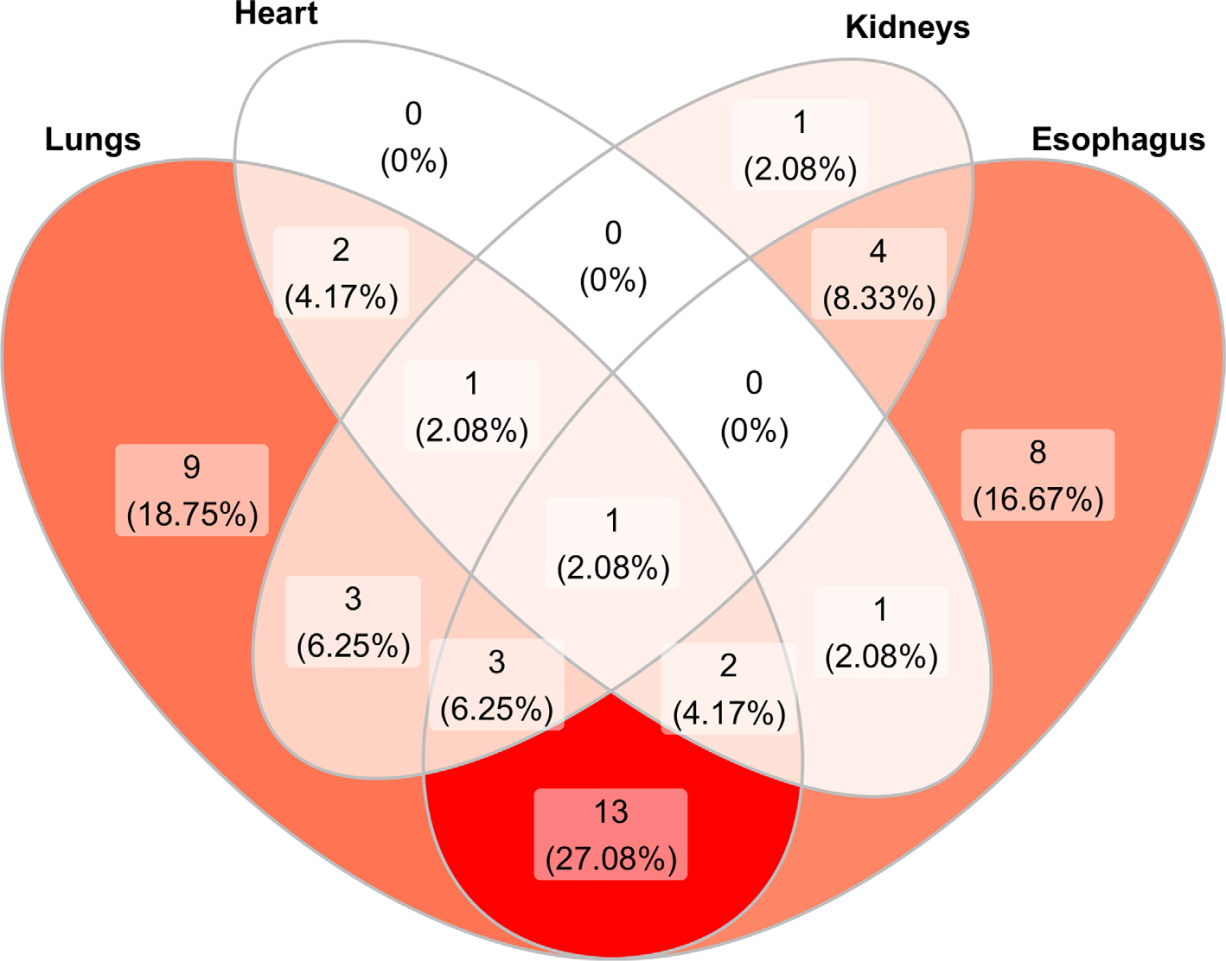
Venn diagram showing the distribution and overlap of organ involvement in the 52 scleroderma patients studied. In dark red color is represented the most common association. The color fades progressively as the association decreases. The majority of patients (27.08%) presented both esophageal and pulmonary involvement while only 1 patient presented all organ involvement. No patient showed only cardiac involvement or kidney–heart or kidney–esophagus involvement.

The overall characteristics are summarized in **Tables 1** **–** **3** and **Figures 1**, **2**. In total, 52 patients were included in the study. Mean ( ± standard deviation) age was 62.02 ( ± 13.3) years, and most of the patients were women (75%, 39/52). A total of 86 complications were recognized in the four investigated sites (mean = 1.8 complications per patient; SD = 0.74).

**Table 1:** Patient characteristics, overall and by organ involvement.

	Overall	Esophagus involvement	Heart involvement	Lungs involvement	Kidney involvement
**N (%)**	52 (100)	32 (61,5)	7 (14,2)	34 (65,3)	13 (25)
**Age (years), Mean (SD)**	62.02 (13.29)	61.56 (13.13)	64.00 (17.30)	62.76 (13.31)	71.85 (11.01)*
**SSc Type, N (%)**					
*Diffused*	18 (34.6)	13 (40.6)	2 (28.6)	13 (38.2)	4 (30.8)
*Limited*	34 (65.4)	19 (59.4)	5 (71.4)	21 (61.8)	9 (69.2)
**Disease duration (years),** **Median [IQR]**	11.5 (7, 8]	9.5 [5, 18]	10 [6, 11.5]	12 [8.25, 18.75]’	12 [10, 18]
**Infusion delay (years),** **Median [IQR]**	3 [1, 8]	2.00 [1.00, 6.25]	0.00 [0.00, 3.50]	3.00 [0.00, 7.00]	5.00 [3.00, 8.00]’
**Rodnan Score, Median [IQR]**	12.50 [5.00, 20.50]	13.50 [5.75, 24.00]‘	14.00 [4.50, 21.00]	15.00 [8.25, 23.00]*	7.00 [4.00, 22.00]’
**ENA Antibodies, N (%)**					
*Anti-Centromere*	22 (42.3)	14 (43.8)	2 (28.6)	13 (38.2)	6 (46.2)
*Anti-Scl70*	17 (32.7)	12 (37.5)	1 (14.3)	12 (35.3)	4 (30.8)
*Anti-RNA polymerase III*	6 (11.5)	4 (12.5)	1 (14.3)	4 (11.8)	0 (0.0)
*Anti-RNP*	3 (5.8)	2 (6.2)	1 (14.3)	3 (8.8)	0 (0.0)
*Anti PM-Scl 100/75*	6 (11.5)	3 (9.4)	3 (42.9)*	5 (14.7)	0 (0.0)
* Anti-SSA/Ro*	18 (34.6)	11 (34.4)	2 (28.6)	12 (35.3)	5 (38.5)

*p < 0.05. ‘p <0.20 for comparisons between patients with or without each organ involvement.

Overall characteristics of the four organs studied. The p values are related to the corresponding negativity groups of the organ studied (not reported in the graph but available in the supplementary appendix). SSc, systemic sclerosis.

**Table 2:** Correlation between overall characteristics, skin and organs involvement (instrumental values: mean +DS) in limited and diffuse cutaneous systemic sclerosis patients.

	Diffused	Limited	p
**N**	18	34	
**Age (years), Mean (SD)**	58.50 (12.90)	63.88 (13.30)	0.167
**Sex (M;F), N**	3;5	10;24	0.312
**Disease duration (years),** **Median [IQR]**	8.00 [5.00, 12.75]	12.00 [9.00, 18.00]	0.108
**Infusion delay (years),** **Median [IQR]**	2.50 [0.25, 6.50]	3.00 [1.00, 8.00]	0.56
**Rodnan Score,** **Median [IQR]**	21.00 [9.75, 26.50]	9.50 [4.25, 15.00]	0.012
**Hypotonic LES, N (%)**	11 (61.1)	11 (32.4)	0.089
**BODY (%)**			
*Minimal hypokinetic alterations*	2 (11.1)	9 (26.5)	0.021
*Dermatomyositis*	1 (5.6)	0 (0.0)	
*Hyperkinetic*	3 (16.7)	0 (0.0)	
*Normal*	2 (11.1)	11 (32.4)	
*Ineffective peristalsis*	10 (55.6)	14 (41.2)	
**Warrick Score, Mean (SD)**	11.39 (8.36)	6.88 (6.40)	0.03
**FVC, Mean (SD)**	86.78 (18.37)	107.71 (26.12)	0.004
**FEV1 Mean (SD)**	83.17 (17.32)	99.09 (23.89)	0.016
**TLC Mean (SD)**	78.44 (18.00)	96.59 (22.42)	0.005
**DLCO Mean (SD)**	70.50 (23.32)	68.71 (17.24)	0.754
**TAPSE/PASP Mean (SD)**	0.84 (0.18)	0.79 (0.19)	0.388
**ACEi, N (%)**	4 (22.2)	11 (32.4)	
**R.R.I., Mean (SD)**	0.67 (0.06)	0.67 (0.06)	0.957

LES, lower esophageal sphincter; FVC, forced vital capacity; FEV1, Forced expiratory volume in the 1st second; TLC, total lung capacity; DLCO, the carbon monoxide diffusing capacity; PASP, pulmonary arterial systolic pressure; TAPSE, tricuspid annular plane systolic excursion; ACEi, Angiotensin-converting enzyme (ACE) inhibitors; R.R.I., renal resistance index.

**Table 3:** Overall characteristics, instrumental values and skin involvement based on the positivity of a specific antibody studied.

Antibody	anti-Scl-70	anti- centromere	anti-RNA Polymerase III	anti-RNP	PM-Scl 100/75	anti-SSA/Ro
**n**	17	22	6	3	6	18
**Age (years), Mean (SD)**	57.47 (12.25)	67.09 (9.54)*	53.17 (9.20)	58.00 (7.21)	50.50 (9.25)	63.44 (15.71)
**Sex (M;F), N**	3;14	5;17	1;5	0;3	2;4	3;15
**SSc type, N (%)**
*Diffused*	15 (88.2)*	0 (0.0)	3 (50.0)	1 (33.3)	3 (50.0)	6 (33.3)
*Limited*	2 (11.8)	22 (100.0)*	3 (50.0)	2 (66.7)	3 (50.0)	12 (66.7)
**Disease duration (years),** **Median [IQR]**	10.00 [5.00, 4.00]	12.00 [9.50, 17.75]	8.00 [5.50, 11.25]	22.00 [15.00, 22.50]	5.50 [2.50, 7.75]	11.50 [9.25, 18.00]
**Infusion delay (years),** **Median [IQR]**	4.00 [0.00, 7.00]	3.00 [1.00, 8.00]	2.00 [0.25, 7.50]	5.00 [4.00, 8.50]	1.00 [0.00, 2.75]	4.50 [1.00, 7.75]
**Rodnan Score,** **Median [IQR]**	15.00 [9.00, 24.00]	11.00 [5.25, 17.25]	16.00 [10.25, 23.25]	15.00 [15.00, 21.00]	10.00 [4.25, 18.00]	9.50 [5.50, 14.75]
**Hypotonic LES, N (%)**	8 (47.1)	8 (36.4)	3 (50.0)	2 (66.7)	2 (33.3)	10 (55.6)
**BODY, N (%)**						
*Minimal hypokinetic alterations*	2 (11.8)	7 (31.8)	0 (0.0)	0 (0.0)	0 (0.0)	4 (22.2)
*Hyperkinetic*	3 (17.6)	0 (0.0)	0 (0.0)	0 (0.0)	1 (16.7)	1 (5.6)
*Normal pattern*	2 (11.8)	4 (18.2)	3 (50.0)	1 (33.3)	3 (50.0)	4 (22.2)
*Ineffective peristalsis*	10 (58.8)	11 (50.0)	3 (50.0)	2 (66.7)	2 (33.3)	9 (50.0)
**Warrick Score, Mean (SD)**	10.79 (8.68)*	4.00 (3.74)	9.50 (6.47)	5.33 (9.24)	14.33 (8.64)*	9.53 (9.39)
**FVC Mean (SD)**	89.65 (23.29)	113.36 3.39)*	103.17 (23.92)	83.67 (28.73)	87.33 (26.14)	97.83 (28.85)
**FEV1 Mean (SD)**	84.65 (20.59)	102.23 (21.62)*	102.17 (21.03)	72.33 (25.48)	78.83 (19.00)	91.33 (26.61)
**TLC Mean (SD)**	78.65 (18.84)	101.05 (20.95)*	93.67 (25.48)	86.67 (33.72)	81.33 (22.23)	87.89 (26.83)
**DLCO Mean (SD)**	70.94 (23.86)	71.45 (16.71)	63.33 (24.62)	58.00 (18.36)	67.50 (8.48)	62.89 (20.02)
**TAPSE/PASP Mean (SD)**	0.83 (0.17)	0.82 (0.21)	0.90 (0.23)	0.66 (0.08)	0.75 (0.21)	0.85 (0.19)
**ACEi, N (%)**	4 (23.5)	5 (22.7)	0 (0.0)	2 (66.7)	2 (33.3)	4 (22.2)
**R.R.I., Mean (SD)**	0.67 (0.06)	0.68 (0.06)	0.63 (0.04)	0.65 (0.05)	0.65 (0.03)	0.67 (0.07)

The p values are related to the corresponding negativity groups of the antibody studied (not reported in the graph but available in the supplementary appendix). *p < 0.05.

Mean age was significantly higher in patients with worse kidney perfusion, compared to the mean age of patients without kidney complications (71.85 *vs* 58.74, OR = 1.10; 95%CI = 1.04–1.19; p = 0.0051) and disease duration was slightly lower in patients with heart complications (10 years) compared to patients without (12 years). Eighteen patients (34.6%) were classified in the dcSSc group and 34 (65.5%) in the lcSSc group. The Rodnan score went from a minimum of 1 to a maximum of 44 (median 12.50, IQR 5.00, 20.50) with a median score of 21.0 and 9.50 for the dcSSc group and lcSSc group respectively (p <0.01). Forty-eight out of 52 patients had at least other organ involvement. Namely, 34 out of 52 (65%) patients presented a pulmonary involvement; 32 (61,5%) patients presented an esophageal impairment; 13 (25%) patients presented a kidney involvement; seven (13,4%) patients presented a cardiological involvement. The esophagus and lung were found to be the most associated organs (13 patients out 52, 27.08%) (**Figure 1**).

Patients with lung complications had significantly higher Rodnan score (mean 15) than patients without (mean 7.5) (OR = 1.09; 95%CI = 1.02–1.18; p = 0.0274). In particular 13 out of 18 dcSSc patients and 21 out of 34 lcSSc patients showed pulmonary abnormalities (DLCO <75%). DcSSc patients showed lower mean FEV1, FVC and TLC values than lcSSc patients [83.1% *vs* 99% (p <0.05)], 86.7% *vs* 107% (p <0.01), 78.4% *vs* 96.5% (p <0.01)], respectively. Median Warrick’s scores were 6.88 for lcSSc group and 11.39 for dcSSc group (p <0.05). Furthermore, patients with lung involvement had a lower mean LES pressure value compared to patients without lung impairment (13.01 mmHg *vs* 20.77 mmHg, p <0.01).

We found a correlation between skin and lung (functional and radiological) involvement. Those patients who presented a lung fibrotic pattern experienced an alteration of the pression of the lower esophageal sphincter.

Patients with esophagus involvement were 32 (61%) and we could not find any differences of mRSS value respect patient without (mRSS: 13.5 *vs* 11.5 respectively, p = 0.07). A mean distal amplitude wave was 34.6 mmHg among dcSSc patients and 45.21 mmHg among lcSSc patients (p = 0.2). The manometry pattern among SSc group was defined as follow: two dcSSc patients (11.1%) and nine lcSSc patients (26.5%) presented a hypokinetic pattern (p <0.05), 10 dcSSc (55.6%) and 14 (41.2%) lcSSc patients with a sclerodermic pattern (p <0.05), two (11.1%) dcSSc patients and 11 (32.4%) lcSSc patient with a normal pattern. One (5.6%) dcSSc patient showed a dermatomyositis pattern while 3 dcSSc (16.7%) patients with a hyperkinetic pattern. Of these patients, only 22 had a hypotonic LES: 11 dcSSc patients showed a mean pressure of 13.66 mmHg and 11 lcSSc patients had a mean pressure of 16.77 mmHg (p = 0.3). Finally, 28% out of our patients had both an esophagus and pulmonary complication with a mean Rodnan score of 21.15 (**Figure 1**). No differences in mean Warrick’s score value was recorded between patients with esophagus involvement and without (7.9 *vs* 9.2, p = 0.5). Furthermore, patients with a hypotonic LES presented the same mean Warrick’s score value than patients with a normotonic LES (9.1 *vs* 8.0, p = 0.6).

We did not found any specific correlation between skin and esophagus. The manometric ‘‘sclerodermic’’ pattern was observed to be the most representative in dcSSc patients while the “sclerodermic” and “normal pattern” were equally represented in lcSSc patients. Furthermore, we have not observed a correlation between motor alteration of the esophagus and lung.

Patients with cardiological involvement: seven (13%) patients (two dcSSc and five lcSSc) presented a cardiac involvement with a TAPSE/PASP ratio <0.64mm/mmHg. These patients presented a median mRSS value of 14.00 (IQR: 4.50, 21.00) without any significant difference compared to patients without cardiac involvement (12.00, IQR: 5.00, 20.00). We did not observe any significant difference between dcSSc and lcSSc forms.

No correlation has been found between the heart and skin and other organs studied.

Patients with kidney involvement: thirteen (25%) patients (four dcSSc and nine lcSSc) presented kidney perfusion alteration with a median R.R.I ≥0.70 mm/mmHg. No statistical difference was found between dcSSc and lcSSc groups (0.67 mm/mmHg v 0.67 mm/mmHg, p = 0.95). Patients who assume ACE-inhibitors presented a mean R.R.I value similar to the group which did not take ACE-inhibitors (0.68 mm/mmHg *vs* 0.62 mm/mmHg, p = 0.15). Patients with Kidney complications had slightly higher mean delay infusion treatments compared to patients without (5 years and 2 years, p <0.1), while for the other complications non-significant trends were in the opposite direction. Patients with renal impairment had a mean lower TAPSE/PASP ratio than the group without (R.R.I. 0.70 *vs* 0.84 respectively, p <0.05).

No correlation has been found between skin and kidney involvement. The renal perfusion has been found identical both in patients who were taking ACE-inhibitors and in those who were not taking them. The renal perfusion has been observed to be worse in those patients where infusion therapy has been delayed and with a decreased cardiac function.

### Serology

ANA tested positive in all patients; 49 (94%) of them showed a positivity for ENA or scleroderma-associated antibodies. Twenty-two patients (42.3%) were found positive for anti-centromere antibodies, 18 (34.6%) for anti-Ro/SSa, 17 (32.7%) for anti-Scl70, six (11.5%) for anti-RNA polymerase-III, six (11.5%) for anti-PM-Scl 100/75 and three (5.8%) for anti-RNP antibody. Only three patients out of 52 (5,8%) were ENA negative. All patients who tested positive for anti-centromere belonged to the lcSSc group, while these antibodies were not observed in the dcSSc group (p <0.01). Out of 17 anti-Scl-70 positive patients, 15 were dcSSc and two lcSSc (p <0.01). Twelve lcSSc patients showed positivity for Ro/SSa antibodies; six patients tested positive in the dcSSc group (p = 0.8). The anti-RNP antibody positivity was observed in one dcSSc patient and two dcSSc patients (p = 0.9). In addition, the distribution of RNA polymerase III and PM-Scl 100/75 antibodies was the same for both dcSSc and lcSSc groups (three patients out of six for each group, p = 0.4 and p = 0.3 respectively). Concerning the relationship between antibody positivity and organ involvement, the following results were found. About pulmonary involvement, patients positive for anti-centromere have better median FEV1, CVF and CPT than the other group (102% *vs* 87%, p <0.01; 113% *vs* 91%, p <0.01; 101% *vs* 82%, p <0.01, respectively). Mean Warrick’s score was lower in anti-centromere positive patients than in the negative group (4 *vs* 11, p <0.01 respectively). Scl-70 positive patients had lower mean FEV1, CVF and CPT than negative patients (84.6% vs 97.9%, p <0.05, 89.6% *vs* 105.7%, p <0.05: 78.6% *vs* 95%, p <0.01 respectively). In both anti-Scl70 and PM-Scl100/75 positive patients, the mean Warrick’s score was higher than in negative patients (10.7 *vs* 6.8, p <0.05: 14.3 and 7.12, p <0.05 respectively). No differences in median DLCO were observed according to anti-centromere and Scl-70 status. No statistical differences related to the other antibodies were seen. About cardiac involvement, patients with hearth complications were more likely to show anti PM-Scl 100/75 positivity (OR = 10.5, 95%CI = 1.5–77.6, p = 0.0153). There were no statistically significant differences between heart severity and other autoimmunity findings. About esophageal and kidney involvement, no statistically significant differences were observed between positive or negative patients for all studied antibodies.

Anti-Scl-70 and anti-centromere antibodies were the most represented among those studied. The former has been observed to be more represented in patients with a diffuse form of the disease, associated with worsened lung function. On the other hand, the latter has been observed in all patients with a limited form of the disease and associated with better lung function. Anti PM-Scl 100/75 antibodies positivity has been found, in our study, to be associated with pulmonary fibrosis and cardiac involvement.

Results of the model system to value the entity of the risk to develop organ-specific complications.

The accordance between predictions and observation, stratified by site, is reported in **Table 4**. For each organ investigated (Esophagus, Heart, Lungs, Kidney) the model has shown the following results:

- Esophagus: TP value was found in 28 patients (60.4%), FP in seven patients (12.5%), TN in nine patients (20.8%) and FN in four patients (6.3%); k (95% CI): 0.46 (0.19, 0.73)
- Hearth: TP value was found in five patients (10.4%), FP in 0 patients (0%), TN in 41 patients (85.4%) and FN in two patients (4.2%); k (95% CI): 0.81 (0.56, 1.00)
- Lungs: TP value was found in 29 patients (60.4%), FP in five patients (12.5%), TN in nine patients (18.8%) and FN in five patients (10.4%); k (95% CI): 0.50 (0.23, 0.77)
- Kidney: TP value was found in nine patients (18.8%), FP in three patients (6.2%), TN in 32 patients (66.7%) and FN in four patients (8.3%); k (95% CI): 0.62 (0.37, 0.88).

**Table 4:** 2 × 2 contingency table for each organ studied.

Site	Model Prediction	N. of patients with no observed complication	N. of patients with observed complication	Cohen’s Kappa (k) (95% CI)
**Esophagus**	No complication	9 (20.8)	4 (6.3)	0.46 (0.19, 0.73)
	Complication	7 (12.5)	28 (60.4)	
**Heart**	No complication	41 (85.4)	2 (4.2)	0.81 (0.56, 1.00)
	Complication	0 (0)	5 (10.4)	
**Lungs**	No complication	9 (18.8)	5 (10.4)	0.50 (0.23, 0.77)
	Complication	5 (10.4)	29 (60.4)	
**Kidney**	No complication	32 (66.7)	4 (8.3)	0.62 (0.37, 0.88)
	Complication	3 (6.2)	9 (18.8)	

The columns: organ complication predicted by the model. The rows: observed organ complication. Total number of patients: 48/52; four patients were excluded due to the absence of at least one organ complication, a necessary criteria for the development of the statistical model. k <0.2: poor concordance; k between 0.2 and 0.4: modest concordance; between 0.41 and 0.61: moderate concordance; between 0.61 and 0.80: good concordance; >0.80: excellent concordance.

Four patients were not included in the evaluation for the absence of at least one organ complication.

**Figure 3:**
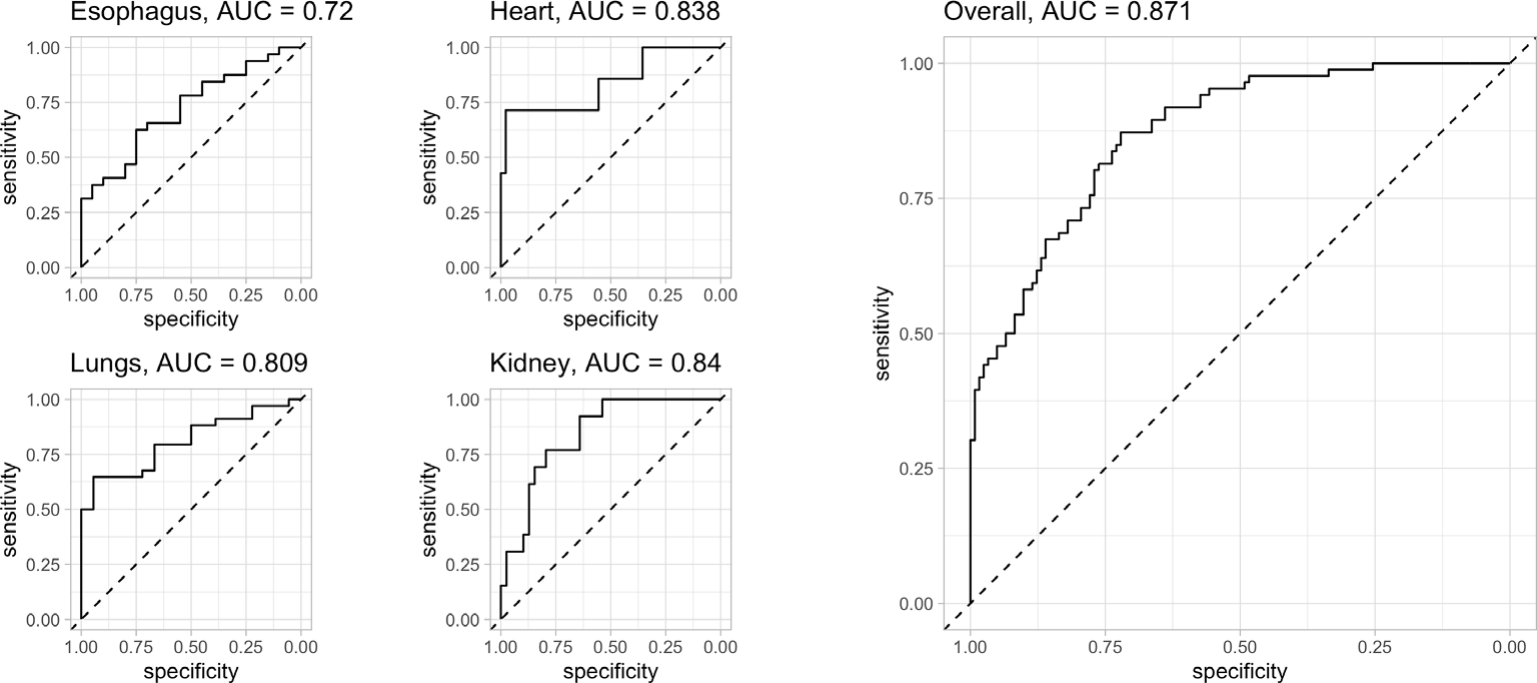
evaluation of the model’s predictive capacity: ROC curve of all the four organs investigated (left side) and overall (right side): for each organ investigated the AUC of esophagus, heart, lung, and kidney were 0.720, 0.838, 0.809 and 0.84 respectively. Combining the four site-specific probability, the final model presents an AUC of 0.871 with an accuracy of 84.4% (95%CI 78, 89), 86% of sensitivity and 83% of specificity. Cohen’s Kappa (k) of 0.68. Legend: AUC = 0.5: no discrimination; AUC: 0.7 to 0.8: acceptable accuracy; AUC: 0.8 to 0.9 excellent accuracy; AUC >0.9 outstanding.

**Figure 4:**
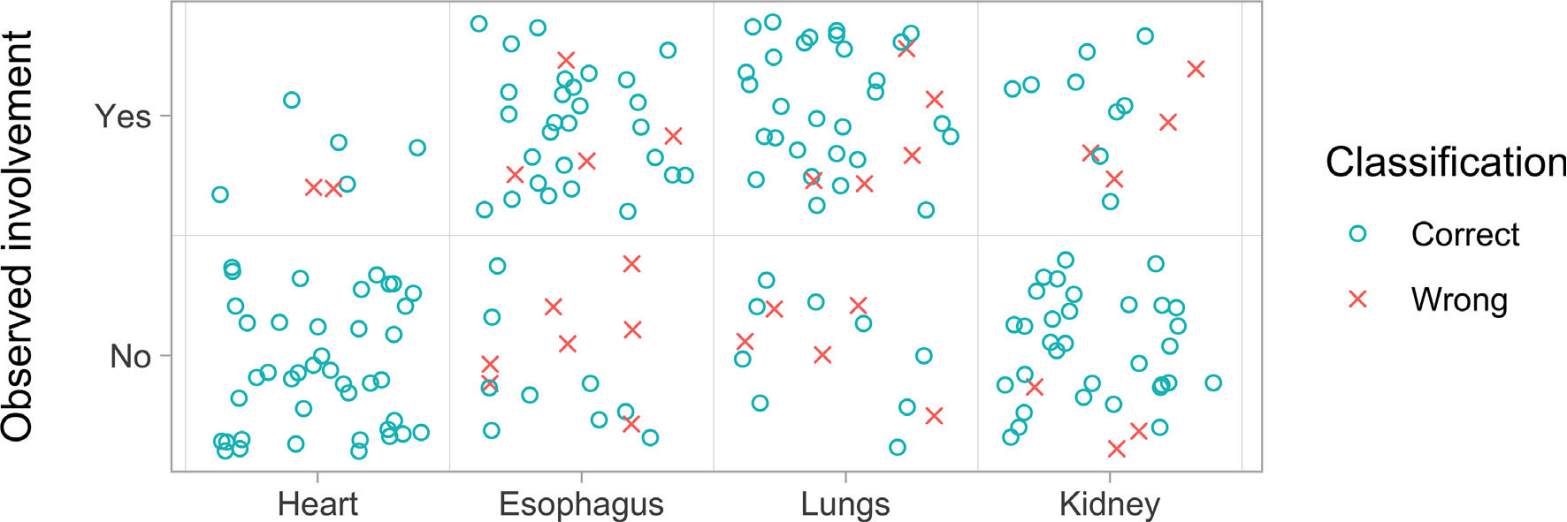
Overview of the prediction model goodness of fit by organ involvement. For each organ investigated are represented the correct (blue circle) and wrong predictions (red x). The data refer to those reported in **Table 4**. For the heart, the model correctly predicted the absence of involvement in all 41 patients and the presence of involvement in seven patients, failing only two cases. For the esophagus, the model predicted the absence of complication in 16 patients and was wrong in seven cases, while it predicted the absence of involvement in 32 cases but was wrong four times. For the lung, the model predicted the absence of involvement in 14 cases and was wrong five times while it predicted the presence of involvement in 34 patients and was wrong five times. Finally, for the kidney, the model predicted the absence of involvement in 35 patients and was wrong three times while it predicted the presence of involvement in 13 patients and was wrong four times.

The prediction capability of each organ-specific complication had been represented by the ROC curve (details in **Figure 3**). The area under the curve (AUC) of the esophagus, heart, lung, and kidney were 0.720, 0.838, 0.809, and 0.84 respectively (**Figure 3**). Combining the four site-specific probabilities, the final model had an accuracy of 84.4% (95%CI 78, 89) in complication-site prediction, AUC of 0.871, 86% of sensitivity, and 83% of specificity, Cohen’s Kappa (k) of 0.68. An overview of the prediction error was summarized in **Figure 4**.

## Discussion

Our patients were mostly female (75%). The mean age was 62 years old. The lcSSc pattern was more represented compared to the dcSSc pattern. The onset of the disease begun later in patients with dcSSc form compared to patients with lcSSc (p <0.05) confirming the latter is an early-onset form with the slow development of the disease. All these findings were supported by the data from the literature (13–15). The relationship between cutaneous involvement (Rodnan score) and the organ complications was variable. The strongest correlation has been observed between the skin and lungs (p <0.001). Several studies support these findings (16–18): in particular, Matsuda et al. (18) described the relationship between skin and lung fibrosis along three aspects: chronological (both develop in the first few years of the natural course of SSc), pathohistological (both share some part of the mechanism that lead to the fibrosis), and therapeutic (B cell-targeting therapy improve skin fibrosis and lung functions). We did not found a correlation between the degree of skin and esophageal involvement. As reported in the literature, there is not a clear relationship: the majority of prior research reports an absence of correlation while few describe it (19). Instead, we found differences in manometric findings in SSc type. In fact, dcSSc presented motor alterations of the esophagus, mostly ineffective peristalsis, while lcSSc patients did not show a prevalent specific pattern. Regarding kidney/heart complications and skin, we did not find any statistically significant correlation. In our study, lcSSc patients, presenting an inflammatory pulmonary pattern, showed a better lung functionality than the dcSSc patients, who had the fibrotic pattern (p <0.01); these findings were provided by spirometer parameters and radiological data, while DLCO values appear to be influenced by both heart and lung function abnormalities and thus non sufficiently specific as an indicator for lung disease. Hence, we underline the importance to utilize all pulmonary diagnostic methods to investigate pulmonary involvement in systemic sclerosis. Furthermore, we think that Warrick’s score is a useful tool to assess the pulmonary severity in scleroderma patients. In fact, it not only describes the structural involvement of the lung but, as we expected, it inversely correlates with the main spirometry parameters and the DLCO. At this point, some considerations are necessary regarding the correlation between lung and esophagus. Our data found 28% of the studied patients had both pulmonary and esophageal complications. These patients showed the worst mean mRSS score (value of 21.5) among all the categories analyzed. This relationship could be explained considering the gastro-esophageal reflux, due to a hypotonic LES, to be responsible for the passage of acidic material in the respiratory tract, which contributes to pulmonary fibrosis (20). Most patients of our cohort who showed an alteration in the esophageal body motility also had an alteration of LES pressure. In addition, patients with pulmonary function impairment presented impaired LES pressure. Unexpectedly, we did not observe differences in the Warrick’s score values between patients with hypotonic and normotonic LES. Probably this result is due to the low number of patients studied and data should be analyzed and compared to a 24-hour pH-impedance study, as reported by Savarino et al. (21)

Interestingly, patients with renal impairment had a mean lower TAPSE/PASP ratio than the group without (p <0.05). We did not find in literature any kind of relationship between these and further investigations are needed. TAPSE/PASP ratio could then be used to assess the renal hemodynamics alterations and the risk of progression of kidney damage. About the relationship between ACE inhibitors and kidney involvement, the intake of ACE-inhibitors has been shown a positive influence on renal dynamics, reducing the cut-off value by 0.70 mm/mmHg in the group taking the drug, without significant differences with the group not taking the drug. The use of this class of drugs has proven effective in modifying renal dynamics by preventing progression to acute renal failure (22). In general, patients with renal involvement were older than the group without. This difference has been found only for this affected organ. It is known that age negatively affects renal perfusion (23) and, probably, in scleroderma patients this event is more pronounced.

Regarding serology, we did not find any correlation between skin involvement and antibody profile. Patients with anticentromere antibody profile, all belonging to the lcSSc group, had a lower degree of lung fibrosis and presented better spirometric values than the negative ones (p <0.01), as expected with data from the literature (24). Conversely, Scl-70 positive and PM-Scl 100/75 positive patients were those who presented the worst degree of lung fibrosis (Warrick’s score of 10.79 and 14.33 respectively). Furthermore, Scl-70 positive patients had a worse pulmonary function (lower FEV1, FVC, and TLC than the negative group). This data confirms what has been described in the literature, namely that positivity for the Scl-70 antibody is associated with a worse risk of pulmonary involvement while anti-centromeric antibody not (17, 25). Regarding PM-Scl 100/75, positive patients had a lower risk of peripheral vasculopathy, pulmonary hypertension, and esophageal involvement, higher risk of pulmonary fibrosis (26). Our study is partly in agreement with these results since we found an association with lung fibrosis (p <0,05) but no with esophageal involvement. In our study, 43% of patients with cardiac involvement tested also positive for PM-Scl 100/75. Although a limited number of patients tested positive for this antibody, we can consider this data significant. In fact, a higher incidence of the PM-Scl 100/75 antibody has been reported in the literature in scleroderma patients with cardiac involvement (27).

We want to emphasize that data from trans-thoracic echocardiography was used only to evaluate the RV function. In daily practice, this methodology is also performed to evaluate the risk of pulmonary hypertension (PH), one of the major complications in sclerodermic patients that lead to RV dysfunction. Right heart catheterization (RHC) is the gold standard for the diagnosis and classification of pulmonary hypertension (28) but only four out of 52 of our patients performed this due to its invasiveness and data were not included in this study.

Finally, our risk prediction model had an excellent accuracy for the identification of organ-specific complications (AUC of 0.871, sensitivity: 86%, specificity 83%, concordance Cohen’s Kappa 0.68). For each organ, accuracy in discriminating patients with and without disease involvement was good or excellent, with AUC ranging from 0.72 to 0.84 (**Figure 3**). These results encourage the feasibility of complication prediction in clinical practice and we believe that this model should be combined with the clinical evaluation of the patient, to identify prematurely those patients who may develop organ damage and this may have a large impact on how the care assistance could be tailored on each specific patients. In an ideal setting, the model could be distributed as a tool for physicians and updated as new evidence emerges to detect and possibly limit the development of organ complications by routing the patient toward the most appropriate specialist. Our results are encouraging, and the model will be further validated in the future based on data from new scleroderma patients to improve its reliability.

This study has some limitations, such as the retrospective data collection; the small sample size, and the missing validation of the predictive performance that should be tested on a different set of data, therefore the project has only an exploratory nature. The strengths of this study are the multi-disciplinary approach and the support to the personalized medicine, since the appropriate predictions may have several clinical practice implications, including resource optimization and early complication detection.

## Conclusion

Retrospective observation of Italian SSc patients followed in our hospital revealed a significant correlation between skin thickness and pulmonary function/imaging. Several results will be the subject of further studies, in particular the cardiac involvement in SSc patients with PM-Scl 100/75 antibody positivity and the evaluation of renal function in SSc patients using the R.R.I. value. The predictive model has proved effective in predicting the risk of developing organ complications but more validations are necessary.

## Data Availability Statement

The raw data supporting the conclusions of this article will be made available by the authors, without undue reservation.

## Ethics Statement

The studies involving human participants were reviewed and approved by Comitato Etico Ospedale Policlinico San Martino. The patients/participants provided their written informed consent to participate in this study.

## Author Contributions

EC idealized and developed the project and writing and revision of the article. AM collected all the data, contributed to the conception and writing of the article, and contributed to the dermatological evaluation. GM contributed to the immunological evaluation and to the writing and revision of the article. MB contributed to the immunological evaluation, contributed to the design and writing of the project, and contributed to the collection of all data. SC contributed to the analysis of CT scans and to the writing of the specific part. PZ contributed to the gastroeneterological; gastroenterological evaluation and participated in the writing and revision of the article. PA contributed to the cardiological evaluation and participated in the writing and revision of the article. MG contributed to the pneumological evaluation and participated in the writing and revision of the article. RR contributed to the nephrological evaluation of and participated in the writing and review of the article. LC contributed to the statistical evaluation of the collected data, developed the predictive model, and contributed to the writing and review of the article. AP reviewed the project and contributed to the writing of the article. All authors contributed to the article and approved the submitted version.

## Supplementary Material

The Supplementary Material for this article can be found online at: https://www.frontiersin.org/articles/10.3389/fimmu.2021.588753/full#supplementary-material

## Conflict of Interest

The authors declare that the research was conducted in the absence of any commercial or financial relationships that could be construed as a potential conflict of interest.
