# Supplementary material for: Correlation Between Skin and Affected Organs in 52 Sclerodermic Patients Followed in a Diseases Management Team: Development of a Risk Prediction Model of Organ-Specific Complications
Source: Front Immunol. 2021 Jun 2;12:588753. doi: 10.3389/fimmu.2021.588753 (PMC8207468; doi:10.3389/fimmu.2021.588753)

## Supplementary appendix

**Table S1:** Overall characteristics of the 4 organs studied (complication: yes; no). For each of these, the presence or absence of complication is indicated (yes; no). The clinical and immunophenotypic characteristics (SSC form and immunological profile) and the instrumental parameters used (mean value + DS) are evaluated within each organ. SSc: systemic sclerosis; LES: lower esophageal sphincter; FVC: forced vital capacity; FEV1: Forced expiratory volume in the 1st second; TLC: total lung capacity; DLCO: the carbon monoxide diffusing capacity; PASP: pulmonary arterial systolic pressure: TAPSE: tricuspid annular plane systolic excursion; ACEi: Angiotensin-converting enzyme (ACE) inhibitors; R.R.I.: renal resistance index.
**: p <0.05. **: p< 0.001. ‘: p< 0.20 for comparisons between patients with or without each organ involvement.*

| | **Overall, N = 52** | **Esophageal involvement** | | **Cardiac involvement** | | **Pulmonary involvement** | | **Renal involvement** | |
|---|---|---|---|---|---|---|---|---|---|
| | | **No, N = 20** | **Yes, N = 32** | **No, N = 45** | **Yes, N = 7** | **No, N = 18** | **Yes, N = 34** | **No, N = 39** | **Yes, N = 13** |
| **Age (Years), Mean (SD)** | 62.02 (13.29) | 62.75 (13.86) | 61.56 (13.13) | 61.71 (12.78) | 64.00 (17.30) | 60.61 (13.53) | 62.76 (13.31) | 58.74 (12.44) | 71.85 (11.01) * |
| **Sex (M;F), N** | 13;39 | 7;13 | 6;26 | 10;35 | 3;4 | 5;13 | 8;26 | 8;31 | 5;8 |
| **SSc Type, N (%)** | | | | | | | | | |
| **Diffused** | 18 (34.6) | 5 (25.0) | 13 (40.6) | 16 (35.6) | 2 (28.6) | 5 (27.8) | 13 (38.2) | 14 (35.9) | 4 (30.8) |
| **Limited** | 34 (65.4) | 15 (75.0) | 19 (59.4) | 29 (64.4) | 5 (71.4) | 13 (72.2) | 21 (61.8) | 25 (64.1) | 9 (69.2) |
| **Disease duration (years), Median [IQR]** | 11.5 [7, 18] I | 12 [10.5, 17.25] | 9.5 [5, 18] | 12 [7, 18] | 10 [6, 11.5] | 8.5 [5.25, 14.25] | 12 [8.25, 18.75]’ | 11 [6.5, 17.5] | 12 [10, 18] |
| **Infusion delay (years), Median [IQR]** | 3 [1, 8] | 4.00 [0.75, 9.25] | 2.00 [1.00, 6.25] | 3.00 [1.00, 8.00] | 0.00 [0.00, 3.50] | 5.50 [1.00, 8.00] | 3.00 [0.00, 7.00] | 2.00 [0.00, 7.00] | 5.00 [3.00, 8.00]’ |
| **Rodnan Score, Median [IQR]** | 12.50 [5.00, 20.50] | 11.00 [4.75, 15.00] | 13.50 [5.75, 24.00] ‘ | 12.00 [5.00, 20.00] | 14.00 [4.50, 21.00] | 7.50 [4.00, 11.50] | 15.00 [8.25, 23.00] * | 14.00 [7.50, 20.00] | 7.00 [4.00, 22.00]’ |
| **ENA Antibodies, N (%)** | | | | | | | | | |
| **Anti-Centromere** | 22 (42.3) | 8 (40.0) | 14 (43.8) | 20 (44.4) | 2 (28.6) | 9 (50.0) | 13 (38.2) | 16 (41.0) | 6 (46.2) |
| **Anti-Scl70** | 17 (32.7) | 5 (25.0) | 12 (37.5) | 16 (35.6) | 1 (14.3) | 5 (27.8) | 12 (35.3) | 13 (33.3) | 4 (30.8) |
| **Anti-RNA polymerase III** | 6 (11.5) | 2 (10.0) | 4 (12.5) | 5 (11.1) | 1 (14.3) | 2 (11.1) | 4 (11.8) | 6 (15.4) | 0 (0.0) |
| **Anti-RNP** | 3 (5.8) | 1 (5.0) | 2 (6.2) | 2 (4.4) | 1 (14.3) | 0 (0.0) | 3 (8.8) | 3 (7.7) | 0 (0.0) |

| | | | | | | | | | |
|---|---|---|---|---|---|---|---|---|---|
| **Anti PM-Scl 100/75** | 6 (11.5) | 3 (15.0) | 3 (9.4) | 3 (6.7) | 3 (42.9) * | 1 (5.6) | 5 (14.7) | 6 (15.4) | 0 (0.0) |
| **Anti-SSA/Ro** | 18 (34.6) | 7 (35.0) | 11 (34.4) | 16 (35.6) | 2 (28.6) | 6 (33.3) | 12 (35.3) | 13 (33.3) | 5 (38.5) |
| | | | | | | | | | |
| **Hypotonic LES, N (%)** | 22 (42.3) | 1 (5.0) | 21 (65.6) * | 18 (40.0) | 4 (57.1) | 7 (38.9) | 15 (44.1) | 16 (41.0) | 6 (46.2) |
| **LES pressure mmHg Mean (SD)** | 15.69 (11.27) | 18.91 (9.96) | 13.68' (11.72) | 16.05 (11.85) | 13.40 (6.61) | 20.77 (13.49) | 13.01 (9.01) * | 16.40 (11.34) | 13.58 (11.23) |
| **Ineffective peristalsis, N (%)** | 24 (46.2) | 0 (0.0) | 24 (75.0) ** | 22 (48.9) | 2 (28.6) | 9 (50.0) | 15 (44.1) | 17 (43.6) | 7 (53.8) |
| **Distal Amplitude Waves Mean (SD)** | 41.55 (29.02) | 63.25 (24.25) | 27.98 (23.06) ** | 39.61 (28.41) | 54.00 (32.15) | 40.72 (31.50) | 41.99 (28.11) | 45.14 (31.15) | 30.77 (18.44)' |
| | | | | | | | | | |
| **Warrick Score, Mean (SD)** | 8.04 (7.52) | 9.20 (8.24) | 7.96 (6.88) | 7.10 (6.72) | 14.50 (10.05) | 4.70 (4.01) | 10.41 (8.02) ** | 7.47 (7.18) | 9.91 (8.63) |
| **FVC Mean (SD)** | 100.46 (25.59) | 101.25 (24.63) | 99.97 (26.55) | 99.56 (26.29) | 106.29 (21.23) | 110.50 (17.72) | 95.15 (27.68) * | 99.28 (26.50) | 104.00 (23.28) |
| **FEV1 Mean (SD)** | 93.58 (22.98) | 96.40 (22.89) | 91.81 (23.22) | 92.29 (22.42) | 101.86 (26.64) | 100.72 (9.40) | 89.79 (26.98)' | 92.56 (24.19) | 96.62 (19.44) |
| **TLC Mean (SD)** | 90.31 (22.57) | 93.95 (24.84) | 88.03 (21.11) | 88.84 (23.18) | 99.71 (16.44) | 100.11 (15.15) | 85.12 (24.26) * | 89.72 (22.15) | 92.08 (24.63) |
| **DLCO Mean (SD)** | 69.33 (19.35) | 71.55 (20.91) | 67.94 (18.52) | 70.87 (19.50) | 59.43 (16.14)' | 88.67 (13.16) | 59.09 (13.32) ** | 69.33 (20.77) | 69.31 (15.00) |
| | | | | | | | | | |
| **TAPSE/PASP Mean (SD)** | 0.81 (0.19) | 0.83 (0.20) | 0.80 (0.18) | 0.85 (0.17) | 0.56 (0.07) ** | 0.83 (0.17) | 0.80 (0.19) | 0.84 (0.20) | 0.70 (0.10) * |
| | | | | | | | | | |
| **ACEi, N (%)** | 15 (28.8) | 6 (30.0) | 9 (28.1) | 13 (28.9) | 2 (28.6) | 4 (22.2) | 11 (32.4) | 9 (23.1) | 6 (46.2) |
| **R.R.I., Mean (SD)** | 0.67 (0.06) | 0.67 (0.06) | 0.67 (0.05) | 0.67 (0.06) | 0.67 (0.04) | 0.66 (0.06) | 0.67 (0.05) | 0.64 (0.03) | 0.74 (0.04) ** |

**Table S2:** Associations between overall characteristics, immunological profile, skin and organs involvement (instrumental values: mean +DS ) and type of cutaneous systemic sclerosis patients (limited or diffuse). SSc: systemic sclerosis; LES: lower esophageal sphincter; FVC: forced vital capacity; FEV1: Forced expiratory volume in the 1st second; TLC: total lung capacity; DLCO: the carbon monoxide diffusing capacity; PASP: pulmonary arterial systolic pressure: TAPSE: tricuspid annular plane systolic excursion; ACEi: Angiotensin-converting enzyme (ACE) inhibitors; R.R.I.: renal resistance index.

| | **Diffused** | **Limited** | **p** |
|---|---|---|---|
| **n** | **18** | **34** | |
| **Age (years), Mean (SD)** | 58.50 (12.90) | 63.88 (13.30) | 0.167 |
| **Sex (M;F), N** | 3;5 | 10;24 | 0.312 |
| **Disease Duration (years), Median [IQR]** | 8.00 [5.00, 12.75] | 12.00 [9.00, 18.00] | 0.108 |
| **Infusion delay (years), Median [IQR]** | 2.50 [0.25, 6.50] | 3.00 [1.00, 8.00] | 0.56 |
| **Rodnan Score, Median [IQR]** | 21.00 [9.75, 26.50] | 9.50 [4.25, 15.00] | 0.012 |
| **Antibodies, N (%)** | | | |
| **Anti-Centromere** | 0 (0.0) | 22 (64.7) | <0.001 |
| **Anti-Scl70** | 15 (83.3) | 2 (5.9) | <0.001 |
| **Anti-RNA polymerase III** | 3 (16.7) | 3 (8.8) | 0.699 |
| **Anti-RNP** | 1 (5.6) | 2 (5.9) | 0.999 |
| **Anti PM-Scl 100/75** | 3 (16.7) | 3 (8.8) | 0.699 |
| **Anti-SSA/Ro** | 6 (33.3) | 12 (35.3) | 0.999 |
| | | | |
| **Ipotonic LES, N (%)** | 11 (61.1) | 11 (32.4) | 0.089 |
| **LES pressure mmHg Mean (SD)** | 13.66 (12.10) | 16.77 (10.84) | 0.348 |
| **BODY (%)** | | | |
| **Minimal hypokinetic alterations** | 2 (11.1) | 9 (26.5) | 0.021 |
| **Dermatomyositis** | 1 (5.6) | 0 (0.0) | |
| **Hyperkinetic** | 3 (16.7) | 0 (0.0) | |
| **Normal pattern** | 2 (11.1) | 11 (32.4) | |
| **Ineffective peristalsis** | 10 (55.6) | 14 (41.2) | |
| **Distal Amplitude Waves Mean (SD)** | 34.64 (33.12) | 45.21 (26.40) | 0.215 |
| | | | |
| **Warrick Score, Mean (SD)** | 11.39 (8.36) | 6.58 (6.40) | 0.03 |
| **FVC Mean (SD)** | 86.78 (18.37) | 107.71 (26.12) | 0.004 |
| **FEV1 Mean (SD)** | 83.17 (17.32) | 99.09 (23.89) | 0.016 |
| **TLC Mean (SD)** | 78.44 (18.00) | 96.59 (22.42) | 0.005 |
| **DLCO Mean (SD)** | 70.50 (23.32) | 68.71 (17.24) | 0.754 |
| | | | |

| | | | |
|---|---|---|---|
| **PASP<br>Mean (SD)** | 29.89 (6.47) | 30.12 (5.87) | 0.898 |
| **TAPSE<br>Mean (SD)** | 24.28 (4.11) | 23.09 (3.80) | 0.302 |
| **TAPSE/PASP<br>Mean (SD)** | 0.84 (0.18) | 0.79 (0.19) | 0.388 |
| **ACEi,<br>N (%)** | 4 (22.2) | 11 (32.4) | |
| **R.R.I.,<br>Mean (SD)** | 0.67 (0.06) | 0.67 (0.06) | 0.957 |
| | | | |
| **Esophagus Complications,<br>N (%)** | 13 (72.2) | 19 (55.9) | 0.394 |
| **Lungs complications,<br>N (%)** | 13 (72.2) | 21 (61.8) | 0.654 |
| **Kidney complications,<br>N (%)** | 4 (22.2) | 9 (26.5) | 0.999 |
| **Heart complications,<br>N (%)** | 2 (11.1) | 5 (14.7) | 0.999 |
| **N. of complications,<br>Mean (SD)** | 1.78 (0.88) | 1.59 (0.86) | 0.455 |
| **No complications,<br>N (%)** | 1 (5.6) | 3 (8.8) | 0.999 |

**Table S3:** Associations between the various immunological profiles (Antibody type: yes; no) and the general, skin and organ characteristics examined (mean value +DS). SSc: systemic sclerosis; LES: lower esophageal sphincter; FVC: forced vital capacity; FEV1: Forced expiratory volume in the 1st second; TLC: total lung capacity; DLCO: the carbon monoxide diffusing capacity; PASP: pulmonary arterial systolic pressure: TAPSE: tricuspid annular plane systolic excursion; ACEi: Angiotensin-converting enzyme (ACE) inhibitors; R.R.I.: renal resistance index.

**: p <0.05. **: p< 0.001. ‘: p< 0.20 for comparisons between patients with or without each antibody positivity.*

| Antibody (Negative/Positive) | anti-Scl70 | | anti-Centromere | | anti-RNA polymerase III | | anti-RNP | | PM-Scl 100/75 | | anti-SSA/Ro | |
|---|---|---|---|---|---|---|---|---|---|---|---|---|
| | - | + | - | + | - | + | - | + | - | + | - | + |
| **n** | 35 | 17 | 30 | 22 | 46 | 6 | 49 | 3 | 46 | 6 | 34 | 18 |
| **Age (years), Mean (SD)** | 64.23 (13.38) | 57.47 (12.25) | 58.30 (14.53) | 67.09 (9.54)* | 63.17 (13.38)* | 53.17 (9.20) | 62.27 (13.58) | 58.00 (7.21) | 63.52 (13.07)* | 50.50 (9.25) | 61.26 (12.01) | 63.44 (15.71) |
| **Sex (M;F), N** | 10;25 | 3;14 | 8;22 | 5;17 | 12;34 | 1;5 | 13;36 | 0;3 | 11;35 | 2;4 | 10;24 | 3;15 |
| **SSc type, N (%)** | | | | | | | | | | | | |
| **Diffused** | 3 (8.6) | 15 (88.2)* | 18 (60.0) | 0 (0.0) | 15 (32.6) | 3 (50.0) | 17 (34.7) | 1 (33.3) | 15 (32.6) | 3 (50.0) | 12 (35.3) | 6 (33.3) |
| **Limited** | 32 (91.4) | 2 (11.8) | 12 (40.0) | 22 (100.0)* | 31 (67.4) | 3 (50.0) | 32 (65.3) | 2 (66.7) | 31 (67.4) | 3 (50.0) | 22 (64.7) | 12 (66.7) |
| **Disease Duration (years), Median [IQR]** | 12.00 [8.00, 18.00] | 10.00 [5.00, 14.00] | 9.50 [5.00, 17.00] | 12.00 [9.50, 17.75] | 12.00 [7.25, 18.00] | 8.00 [5.50, 11.25] | 11.00 [7.00, 17.00] | 22.00 [15.00, 22.50] | 12.00 [8.00, 18.00] | 5.50 [2.50, 7.75] | 11.50 [5.00, 17.00] | 11.50 [9.25, 18.00] |
| **Infusion delay (years), Median [IQR]** | 3.00 [1.00, 8.00] | 4.00 [0.00, 7.00] | 2.50 [0.00, 7.75] | 3.00 [1.00, 8.00] | 3.00 [1.00, 8.00] | 2.00 [0.25, 7.50] | 3.00 [1.00, 8.00] | 5.00 [4.00, 8.50] | 3.50 [1.00, 8.00] | 1.00 [0.00, 2.75] | 2.50 [0.25, 8.00] | 4.50 [1.00, 7.75] |
| **Rodnan Score, Median [IQR]** | 10.00 [5.00, 18.00] | 15.00 [9.00, 24.00] | 14.50 [5.00, 23.75] | 11.00 [5.25, 17.25] | 12.00 [4.25, 20.00] | 16.00 [10.25, 23.25] | 12.00 [5.00, 20.00] | 15.00 [15.00, 21.00] | 12.50 [5.25, 21.50] | 10.00 [4.25, 18.00] | 15.00 [5.00, 22.00] | 9.50 [5.50, 14.75] |
| **Hypotonic LES, N (%)** | 14 (40.0) | 8 (47.1) | 14 (46.7) | 8 (36.4) | 19 (41.3) | 3 (50.0) | 20 (40.8) | 2 (66.7) | 20 (43.5) | 2 (33.3) | 12 (35.3) | 10 (55.6) |
| **LES pressure mmHg Mean (SD)** | 14.94 (8.72) | 17.25 (15.48) | 15.74 (12.88) | 15.63 (8.93) | 15.94 (11.73) | 13.80 (7.26) | 15.96 (11.53) | 11.40 (4.35) | 15.97 (11.80) | 13.58 (6.11) | 17.15 (12.82) | 12.94 (7.08) |
| **BODY, N (%)** | * | | | | | | | | | | | |
| **Minimal hypokinetic alterations** | 9 (25.7) | 2 (11.8) | 4 (13.3) | 7 (31.8) | 11 (23.9) | 0 (0.0) | 11 (22.4) | 0 (0.0) | 11 (23.9) | 0 (0.0) | 7 (20.6) | 4 (22.2) |
| **Dermatomyositis** | 1 (2.9) | 0 (0.0) | 1 (3.3) | 0 (0.0) | 1 (2.2) | 0 (0.0) | 1 (2.0) | 0 (0.0) | 1 (2.2) | 0 (0.0) | 1 (2.9) | 0 (0.0) |
| **Hyperkinetic** | 0 (0.0) | 3 (17.6) | 3 (10.0) | 0 (0.0) | 3 (6.5) | 0 (0.0) | 3 (6.1) | 0 (0.0) | 2 (4.3) | 1 (16.7) | 2 (5.9) | 1 (5.6) |
| **Normal pattern** | 11 (31.4) | 2 (11.8) | 9 (30.0) | 4 (18.2) | 10 (21.7) | 3 (50.0) | 12 (24.5) | 1 (33.3) | 10 (21.7) | 3 (50.0) | 9 (26.5) | 4 (22.2) |
| **Ineffective peristalsis** | 14 (40.0) | 10 (58.8) | 13 (43.3) | 11 (50.0) | 21 (45.7) | 3 (50.0) | 22 (44.9) | 2 (66.7) | 22 (47.8) | 2 (33.3) | 15 (44.1) | 9 (50.0) |
| **Distal Amplitude Waves (mmHg) Mean (SD)** | 44.40 (26.45) | 35.68 (33.82) | 43.52 (32.67) | 38.86 (23.64) | 40.38 (28.50) | 50.50 (34.24) | 42.19 (28.85) | 31.00 (36.37) | 40.18 (27.66) | 52.00 (39.48) | 40.40 (29.57) | 43.72 (28.66) |
| **Warrick Score, Mean (SD)** | 6.88 (6.78) | 10.79 (8.68)* | 11.04 (8.24)* | 4.00 (3.74) | 7.83 (7.71) | 9.50 (6.47) | 8.23 (7.48) | 5.33 (9.24) | 7.12 (6.99) | 14.33 (8.64)* | 7.20 (6.24) | 9.53 (9.39) |
| **FVC Mean (SD)** | 105.71 (25.30)* | 89.65 (23.29) | 91.00 (23.17) | 113.36 (23.39)* | 100.11 (26.03) | 103.17 (23.92) | 101.49 (25.35) | 83.67 (28.73) | 102.17 (25.30) | 87.33 (26.14) | 101.85 (24.03) | 97.83 (28.85) |
| **FEV1 Mean (SD)** | 97.91 (23.09) * | 84.65 (20.59) | 87.23 (22.17) | 102.23 (21.62)* | 92.46 (23.20) | 102.17 (21.03) | 94.88 (22.45) | 72.33 (25.48) | 95.50 (22.92) | 78.83 (19.00) | 94.76 (21.14) | 91.33 (26.61) |
| **TLC Mean (SD)** | 95.97 (22.27)* | 78.65 (18.84) | 82.43 (20.64) | 101.05 (20.95)* | 89.87 (22.43) | 93.67 (25.48) | 90.53 (22.20) | 86.67 (33.72) | 91.48 (22.58) | 81.33 (22.23) | 91.59 (20.28) | 87.89 (26.83) |
| **DLCO Mean (SD)** | 68.54 (17.08) | 70.94 (23.86) | 67.77 (21.22) | 71.45 (16.71) | 70.11 (18.75) | 63.33 (24.62) | 70.02 (19.37) | 58.00 (18.36) | 69.57 (20.39) | 67.50 (8.48) | 72.74 (18.37) | 62.89 (20.02) |
| **PASP Mean (SD)** | 30.11 (5.97) | 29.88 (6.30) | 30.50 (6.10) | 29.41 (6.00) | 30.35 (5.96) | 27.67 (6.53) | 29.96 (5.95) | 31.33 (8.50) | 29.74 (5.93) | 32.33 (6.77) | 30.53 (5.52) | 29.11 (6.95) |
| **TAPSE Mean (SD)** | 23.23 (3.90) | 24.06 (4.01) | 23.67 (3.85) | 23.27 (4.07) | 23.48 (3.96) | 23.67 (3.88) | 23.69 (3.90) | 20.33 (3.06) | 23.52 (4.10) | 23.33 (2.25) | 23.26 (3.64) | 23.94 (4.47) |
| **TAPSE/PASP Mean (SD)** | 0.80 (0.19) | 0.83 (0.17) | 0.80 (0.17) | 0.82 (0.21) | 0.80 (0.18) | 0.90 (0.23) | 0.82 (0.19) | 0.66 (0.08) | 0.81 (0.18) | 0.75 (0.21) | 0.78 (0.18) | 0.85 (0.19) |
| **ACEi, N (%)** | 11 (31.4) | 4 (23.5) | 10 (33.3) | 5 (22.7) | 15 (32.6) | 0 (0.0) | 13 (26.5) | 2 (66.7) | 13 (28.3) | 2 (33.3) | 11 (32.4) | 4 (22.2) |
| **R.R.I., Mean (SD)** | 0.67 (0.06) | 0.67 (0.06) | 0.67 (0.05) | 0.68 (0.06) | 0.67 (0.06) | 0.63 (0.04) | 0.67 (0.06) | 0.65 (0.05) | 0.67 (0.06) | 0.65 (0.03) | 0.67 (0.05) | 0.67 (0.07) |

Figure S1. Complication risk modeling procedure.

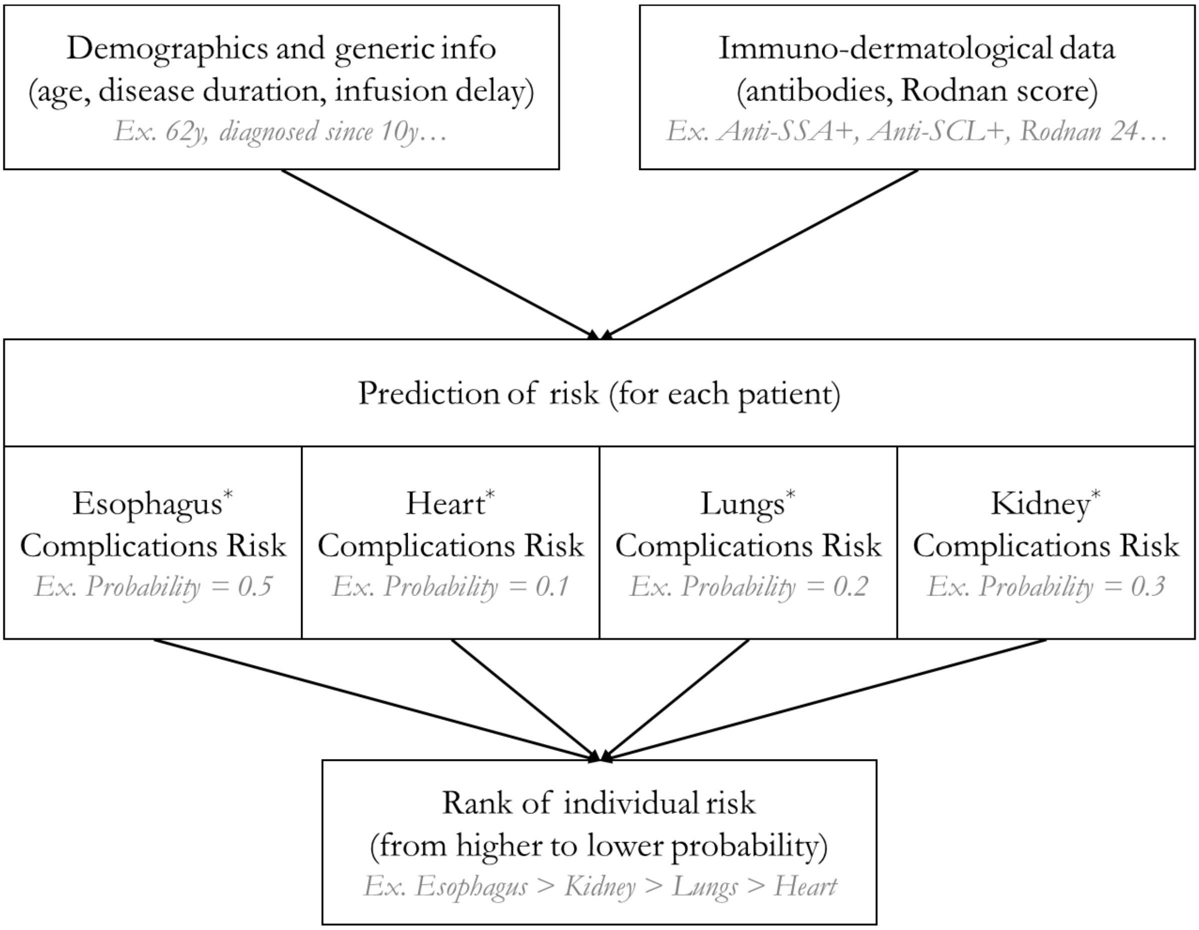

Figure S2. Exemplification of the goodness of fit estimation procedure.

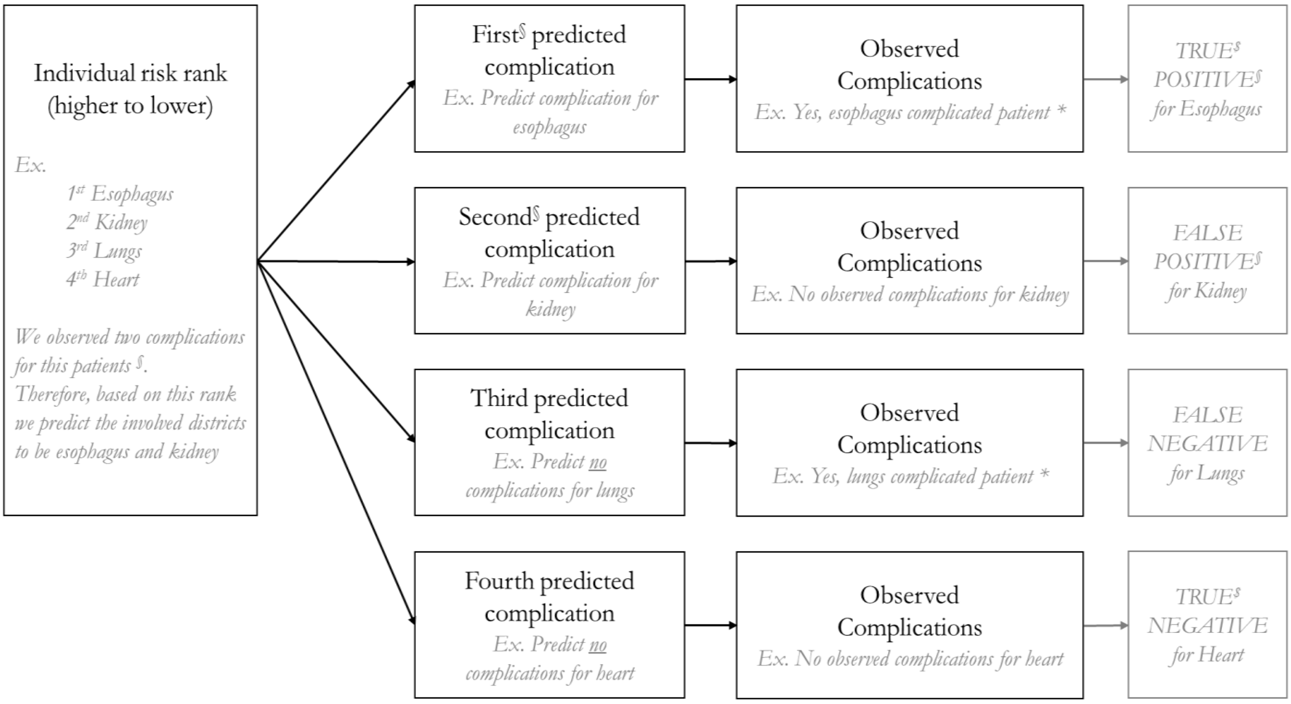

Supplement: Supplementary file 1 [file DataSheet_1.pdf]
